# Hepatitis C Co-infection Among Pregnant Women With Syphilis in West Virginia, 2019–2023

**DOI:** 10.1093/ofid/ofaf716

**Published:** 2025-11-21

**Authors:** Alana G Hudson, Bianca Huff, Jared Boote, Kady Pack, Margret Watkins, Hanna White, Suzanne Wilson, Shannon McBee

**Affiliations:** West Virginia University Cancer Institute, Morgantown, West Virginia, USA; Department of Cancer Prevention and Control, West Virginia University School of Medicine, Morgantown, West Virginia, USA; Bureau for Public Health, West Virginia Department of Health, Charleston, West Virginia, USA; Bureau for Public Health, West Virginia Department of Health, Charleston, West Virginia, USA; Bureau for Public Health, West Virginia Department of Health, Charleston, West Virginia, USA; Bureau for Public Health, West Virginia Department of Health, Charleston, West Virginia, USA; Bureau for Public Health, West Virginia Department of Health, Charleston, West Virginia, USA; Bureau for Public Health, West Virginia Department of Health, Charleston, West Virginia, USA; Bureau for Public Health, West Virginia Department of Health, Charleston, West Virginia, USA; Bureau for Public Health, West Virginia Department of Health, Charleston, West Virginia, USA

**Keywords:** congenital syphilis, hepatitis c, maternal-child health, pregnancy, syphilis

## Abstract

Syphilis and hepatitis C virus (HCV) rates have increased among women of childbearing age. Given the potential for vertical transmission of both infections, we examined syphilis and HCV co-infection among pregnant women in West Virginia (2019–2023). Public health surveillance data for syphilis and HCV were cross-referenced to identify co-infections. Of the 161 pregnant women with syphilis, 42.9% had a past or present HCV infection. Compared with those without HCV, a greater proportion of women with past or present HCV self-reported incarceration (21.7% vs 5.4%) and drug use (50.7% vs 15.2%) during the past year. Notably, women with past or present HCV had lower adherence to syphilis treatment (56.5% vs 84.8%) and a higher frequency of reported congenital syphilis outcomes (59.4% vs 28.3%). Findings support the need for integrated care approaches to address overlapping infections and associated social vulnerabilities.

## BACKGROUND

Syphilis and hepatitis C virus (HCV) can be transmitted from mother to infant, leading to potential serious health issues in children [1, 2]. Rates of syphilis and HCV infections have increased nationwide, with particularly concerning trends observed among individuals of reproductive age [3, 4]. Maternal syphilis rates in the United States more than tripled from 2016 to 2022, then leveled off in 2023 [3, 5]. A similar upward trend has been observed in maternal HCV infections [6, 7]. Consequently, congenital syphilis and perinatal HCV exposures have increased in tandem [3, 4, 6]. In West Virginia, congenital syphilis cases rose from zero in 2015 to 18 in 2023 [3, 8]. While perinatal HCV exposure is not reportable in the state, birth certificate data suggest high exposure rates [9]. A recent population-based study of all individuals who gave birth in West Virginia found a maternal HCV infection rate of 38 per 1000 deliveries [10]. In 2022, the first year of reporting, West Virginia recorded 11 cases of perinatal hepatitis C [11].

Co-occurrence of syphilis and hepatitis C during pregnancy poses a dual threat to maternal and infant health, highlighting a need for effective public health, provider, and health system responses. Both syphilis and HCV are curable; syphilis with antibiotics and HCV with direct-acting antivirals [12, 13]. However, the latter is not currently recommended for use during pregnancy. Universal screening for both infections is recommended during each pregnancy [9, 14]. While syphilis screening during pregnancy has been a longstanding recommendation, universal HCV screening during pregnancy was only first recommended in 2020 [9, 14].

Despite universal screening recommendations, pregnant women who use substances are less likely to be screened for syphilis, and if diagnosed, may be less likely to complete recommended treatment [15]. Syphilis and HCV transmission often occur among people who use drugs, a population that frequently encounters challenges to accessing care and receiving appropriate treatment [11, 16–19]. Substance use during pregnancy is associated with both infections [10, 15]. Incarceration and other social vulnerabilities can hinder access to screening, treatment, and follow-up care, pointing to the need for integrated strategies to prevent and manage infections during pregnancy. Given the rising rates of these infections, understanding the overlap of syphilis and hepatitis C in pregnancy is critical to addressing gaps in care and preventing adverse maternal and infant outcomes. To explore this, we investigated the prevalence of syphilis and HCV co-infection among pregnant women in West Virginia, examined patterns of substance use and syphilis treatment, and assessed outcomes of congenital syphilis.

## METHODS

### Inclusion Criteria

This analysis included all women with a syphilis infection reported to the West Virginia Department of Health (WVDH) through routine public health surveillance between 1 January 2019 and 31 December 2023 who were either pregnant at time of infection or diagnosed in the immediate post-partum period and were residents of West Virginia at time of report. This included individuals diagnosed with syphilis before 2019 whose infection remained untreated or inadequately treated while pregnant in 2019, as well as those with a syphilis infection during pregnancy in 2023 who delivered in 2024. Pregnant women were included if they met standardized criteria based on the Council of State and Territorial Epidemiologists (CSTE) case definition for any stage of syphilis, or if they delivered a stillborn or live-born infant with confirmed or probable congenital syphilis [20]. Records were consolidated into a single entry when multiple syphilis infections were reported during the same pregnancy.

### Data Sources

Syphilis and congenital syphilis are reportable conditions in West Virginia, requiring healthcare providers and laboratories to report cases and positive laboratory test results to the WVDH. Hepatitis C is also a reportable condition, and positive laboratory test results must be reported to WVDH. Negative results for syphilis and HCV are not reportable. Disease Investigation Specialists (DIS) attempt to interview all persons diagnosed with syphilis promptly after case initiation, verify treatment completion, and document follow-up in the West Virginia Electronic Disease Surveillance System (WVEDSS), an integrated information system that houses public health laboratory and case investigation data.

### Variable Classification

Demographic characteristics (age, race), syphilis diagnosis and treatment, past-year incarceration, and past-year substance use were obtained from syphilis case investigation and DIS interview, if not lost to follow-up. Age was defined as the age at the time of the first documented syphilis investigation associated with the pregnancy event. Birth outcomes were classified as live birth, stillbirth, or unknown. Unknown outcomes reflect instances where delivery records could not be verified due to loss to follow-up or out-of-state delivery. Past-year incarceration referred to any self-reported jail or prison time in the 12 months prior to the interview. Past-year substance use was defined as any self-reported use of marijuana, inhalants, crack, cocaine, heroin, methamphetamines, opioids, and benzodiazepines in the 12 months preceding the investigation interview. Syphilis stage at diagnosis was classified using the CSTE surveillance case definition [20]. Adequate syphilis treatment was defined as completion of a CDC-recommended regimen appropriate to the stage of infection, started at least 30 days before delivery in accordance with the CDC's Sexually Transmitted Infections (STI) Treatment Guidelines, applicable at the time of syphilis diagnosis [12, 21]. Treatment adherence was determined at the time the syphilis investigation was closed. For a small subset of cases (n = 3), adherence status could not be verified due to factors such as the complexity of neurosyphilis management, out of state care, and lack of medical records for those lost to follow-up. Rural-urban status was assigned using the United States Department of Agriculture (USDA) 2023 Rural-Urban Continuum Codes, based on the county of residence at time of syphilis investigation [22]. We used 2023 codes exclusively, rather than combining with the earlier 2013 classification, to ensure consistent and up-to-date categorization across study years. Infants with congenital syphilis were classified according to the CSTE case definition [20]. We cross-referenced syphilis and hepatitis C records from WVEDSS using shared unique identifiers to link reports. Past or present HCV infection was defined as ever having a laboratory-confirmed reactive HCV antibody or positive result for HCV RNA or genotype. Confirmed active HCV infection during pregnancy was classified as a positive HCV RNA or genotype test collected during pregnancy or within 2 weeks postpartum. Because universal screening recommendations for HCV during pregnancy were issued after the start of the study period, the primary analyses focus on past or present infection rather than limiting to laboratory-confirmed HCV viremia during pregnancy [9, 23]. Confirmed active HCV infection during pregnancy is briefly noted descriptively in the results; however, detailed data are not shown.

### Data Analysis

Descriptive analyses were performed using frequency distributions. Because the data include the entire population of reported cases meeting inclusion criteria, rather than a sample, inferential statistical tests (eg, *P* values) were not applied. Counts and percentages are presented. Data were analyzed using SAS version 9.4 (SAS Institute, Cary, NC).

## RESULTS

Characteristics and outcomes of pregnant women with syphilis, stratified by past or present HCV, are shown in Table 1. From 2019 to 2023, 161 pregnancies were identified among women with a reported syphilis infection in West Virginia. Overall, 69 (42.9%) had laboratory evidence of a past or present HCV infection; among these, 38 (55.1%) had evidence of a confirmed active HCV infection during pregnancy (Table 1, footnote a). Only 10.1% of women with past or present HCV infection were aged 22 years or younger, compared with 31.5% among those without evidence of past or present HCV infection. More women with past or present HCV identified as White (94.2% vs 80.4%) and resided in non-rural areas (92.8% vs 83.7%). Birth outcomes were documented for most pregnancies; however, 6.2% had unknown outcomes. All recorded stillbirths (n = 3) occurred among individuals with past or present HCV infection.

**Table 1.** Characteristics and Outcomes of Pregnant Women With Syphilis by Hepatitis C Status, West Virginia, 2019–2023

Characteristic	Total Pregnancies (n = 161)	No History of HCV Infection (n = 92)	Past or Present HCV^a^ (n = 69)
Age at syphilis investigation, years			
≤22	36 (22.4)	29 (31.5)	7 (10.1)
23–29	73 (45.3)	37 (40.2)	36 (52.2)
≥ 30	52 (32.3)	26 (28.3)	26 (37.7)
Race			
White	139 (86.3)	74 (80.4)	65 (94.2)
Other	22 (13.7)	18 (19.6)	4 (5.8)
Rurality			
Rural	20 (12.4)	15 (16.3)	5 (7.2)
Non-rural	141 (87.6)	77 (83.7)	64 (92.8)
Reported incarceration in past year			
Yes	20 (12.4)	5 (5.4)	15 (21.7)
No	81 (50.3)	56 (60.9)	25 (36.2)
Unknown	60 (37.3)	31 (33.7)	29 (42.0)
Reported drug use in past year			
Yes	49 (30.4)	14 (15.2)	35 (50.7)
No	57 (35.4)	48 (52.2)	9 (13.0)
Unknown	55 (34.2)	30 (32.6)	25 (36.2)
Syphilis stage of diagnosis			
Primary	10 (6.2)	7 (7.6)	3 (4.3)
Secondary	12 (7.5)	9 (9.8)	3 (4.3)
Early non-primary, non-secondary	43 (26.7)	24 (26.1)	19 (27.5)
Unknown duration or late	96 (59.6)	52 (56.5)	44 (63.8)
Syphilis treatment adherence			
Yes	117 (72.7)	78 (84.8)	39 (56.5)
No	41 (25.5)	13 (14.1)	28 (40.6)
Unknown	3 (1.9)	1 (1.1)	2 (2.9)
Birth outcome			
Live birth	148 (91.9)	85 (92.4)	63 (91.3)
Stillbirth	3 (1.9)	0 (0.0)	3 (4.3)
Unknown	10 (6.2)	7 (7.6)	3 (4.3)
Reported congenital syphilis outcome^b^			
Yes	67 (41.6)^b^	26 (28.3)	41 (59.4)^c^
No	94 (58.4)	66 (71.7)	28 (40.6)

Abbreviation: HCV, hepatitis C virus.

Data are presented as n (%). Percentages may not add up to 100% due to rounding.

^a^Of the 69 individuals with past or present HCV infection, 38 (55.1%) had documented evidence of active infection during pregnancy. For the remaining cases, infection status was unknown due to the absence of universal HCV screening recommendations during much of the study period and because negative test results are not required to be reported under state law.

^b^Congenital syphilis pregnancy outcome includes pregnancy outcomes that meet the CSTE surveillance case definition for syphilitic stillbirth and live-born infants with probable or confirmed congenital syphilis. Outcomes were recorded per pregnancy. In cases of multiple births, if one or more infants were affected the outcome was counted once. This occurred in one twin pregnancy.

^c^Includes n = 2 syphilitic stillbirths.

Among those with past or present HCV, 21.7% self-reported incarceration in the past year, compared with 5.4% of individuals without a documented HCV history. Drug use within the past year was reported by 50.7% of women with past or present HCV infection, compared with 15.2% of those without evidence of infection. Information on past-year incarceration and drug use history was unavailable for approximately one-third of individuals. Most pregnant women (59.6%) with syphilis were staged as unknown duration or late, with a slightly higher proportion among those with past or present HCV (63.8%) compared with those without an HCV history (56.5%). Additionally, adequate syphilis treatment was less common among women with past or present hepatitis C, with 56.5% completing the full treatment regimen compared with 84.8% of those without evidence of hepatitis C. Sixty-seven pregnancies resulted in a reported congenital syphilis outcome, including one twin gestation, which was counted as a single event in the analysis. The percentage of pregnancies resulting in congenital syphilis was higher among women with past or present hepatitis C (59.4% vs 28.3%).

## DISCUSSION

National data show rising syphilis and HCV rates among pregnant women, yet population-level estimates of co-infection are limited [3–7]. We documented past or present HCV infection in 42.9% of pregnant women with syphilis, a group that also more frequently reported recent substance use and less often received adequate syphilis treatment. Despite its importance, few studies have explored syphilis and HCV in pregnancy. A recent analysis of birth certificate data found a strong association between these infections, reporting an adjusted odds ratio of 5.88 [24]. Reported co-infection percentages in the literature range from 7.6% to 21.3% [25–27]. Although we observed a comparatively high percentage of pregnant women with syphilis who had past or present HCV, this may reflect differences in study design, population characteristics, and testing practices. Notably, prior studies have not integrated laboratory-confirmed infections with population-level data, limiting direct comparisons. Additionally, our analysis found that congenital syphilis outcomes were more common among individuals with past or present HCV. This aligns with previous, though limited, research and underscores the need for continued investigation into the maternal and infant health consequences of these intersecting infections [25].

Syphilis is treatable; yet, many pregnant women in our study received inadequate treatment, especially those with evidence of a past or present HCV infection, highlighting the importance of integrating care coordination strategies. Prenatal care should include routine first-trimester syphilis and HCV screening [9, 14]. Given increasing congenital syphilis rates, universal rescreening of syphilis during the third trimester and at birth is recommended [28]. Early detection and treatment of syphilis are crucial to prevent congenital syphilis and ongoing transmission. While HCV treatment is not currently recommended during pregnancy, early identification can facilitate postpartum treatment and infant follow-up [13]. Despite existing guidelines recommending postpartum and infant follow-up care for hepatitis C, several obstacles exist, including limited healthcare access, loss of follow-up, lack of awareness about the importance and timing of infant testing, and insurance-related challenges [29, 30].

Improving maternal and infant outcomes linked to syphilis and HCV requires a coordinated public health response effort. Key strategies include integrating testing for both infections in high-prevalence settings and embedding peer navigators or case managers within prenatal programs and nontraditional settings, including substance use disorder programs and correctional facilities [31–33]. Our findings suggest that individuals with histories of incarceration and recent drug use are disproportionately affected by syphilis and HCV co-infection, emphasizing the need for targeted outreach. Given lower screening and treatment rates among people who use substances, active outreach and tailored services are essential [15]. While point-of-care tests support early intervention, antibody-based tests alone are insufficient to diagnose syphilis or HCV and may have limited utility in settings with a high prevalence of previously treated infections. Expanding the role of DIS to include partner services and linkage to care for individuals with HCV may help reach those who might otherwise remain undiagnosed or untreated.

In summary, this study documented co-occurrence of syphilis and HCV among pregnant women, revealing overlap with substance use and inadequate syphilis treatment. Understanding these relationships may inform strategies to improve maternal and infant care. Limitations include a small sample size and unknown substance use and incarceration history for one-third of cases due to follow-up loss or non-response. Additionally, birth outcomes were unknown for 6.2% of pregnancies, potentially leading to underestimation of congenital syphilis cases. Although similarly distributed by HCV status, unknown values limit the completeness of findings. Including individuals with probable syphilis may have overestimated the disease burden, and classifying HCV infection as both past and present infection limits differentiation between active and resolved infections. Despite limitations, strengths include population-based data and laboratory-confirmed test results, which support the reliability and generalizability of the findings.

## CONCLUSIONS

This study highlights the intersection between syphilis and HCV infection during pregnancy and adds to growing concerns about these rising infections in individuals of reproductive age and infants. Addressing shared drivers, such as substance use and barriers to care, requires a coordinated public health response that supports access to comprehensive care before, during, and after pregnancy, along with sustained efforts to support appropriate infant follow-up.
